# Relationship between sunlight and range use of commercial free-range hens in Australia

**DOI:** 10.1371/journal.pone.0268854

**Published:** 2022-05-31

**Authors:** Md Sohel Rana, Caroline Lee, Jim M. Lea, Dana L. M. Campbell

**Affiliations:** 1 Department of Animal Science, School of Environmental and Rural Science, University of New England, Armidale, NSW, Australia; 2 Agriculture and Food, Commonwealth Scientific and Industrial Research Organisation (CSIRO), Armidale, NSW, Australia; 3 Department of Livestock Services, Ministry of Fisheries and Livestock, Dhaka, Bangladesh; Annamalai University, INDIA

## Abstract

In Australia, summer brings intense, bright sunlight with high ultraviolet (UV) radiation and hot temperatures, which might impact free-range hens’ ranging outside. To determine how range use was correlated with different sunlight variables and weather factors, a study was carried out on three commercial free-range layer farms during the summer/autumn period (December-May) across diverse regions of Australia in Tasmania (TAS), Queensland (QLD), and Western Australia (WA). Hens’ range distribution was determined by counting the number of hens in the direct sunlight (‘sun’) or ‘cloud’ and shaded areas (‘sun-shade’ or ‘cloud-shade’, in sunny or cloudy conditions, respectively) using image snapshots taken at 30 min intervals from video recordings of a portion of one shed comprising 20,000–30,000 hens on each farm during the production phase of the laying cycle. The solar radiation spectrum [UV radiation (UV_AB_) (288–432 nm), photosynthetically active radiation (PAR; visible light) (400–700 nm), and total solar radiation (TSR) (285 nm-3000 nm)] and weather data (ambient temperature and relative humidity) were recorded through an on-site weather station. Data were analysed separately due to discrepancies between the farms’ layouts. The effects of time of day and months on range use were analysed using General Linear Models in JMP^®^ 16.0 and the relationship of sunlight and weather variables with hens’ distribution in ‘sun’/’sun-shade’ and ‘cloud’/’cloud-shade’ in sunny and cloudy conditions respectively was determined by fitting linear ridge regression models using the *‘lmridge’* package in R. Overall, the time of day and month had significant effects on hens’ distribution on the range (all p < 0.0001). Hens’ range occupancy in the ‘sun’ decreased during the midday period with gradual increases in the late afternoon to evening, and the opposite pattern in the ‘sun-shade’. A linear increase in the number of hens on the range over the months indicated the seasonal effects on hen ranging patterns. Temperature, UV_AB_ and PAR were the most important factors for discouraging hens’ range use in the ‘sun’ suggesting free-range systems in Australia should be designed to account for the extreme sunlight using adequate shade for optimum ranging across summer.

## Introduction

Free-range poultry systems are prevalent within Australia and consumers favour them due to their perceived greater naturalness. Free-range laying hen systems in place of intensive indoor housing meet consumer demands of perceived improved welfare as well as perceived healthier eggs [1–4]. Free-range hens get exposure to sunlight for at least part of the day with access to move freely in an outdoor area rather than continuous indoor housing. The outdoor exposure provides hens the opportunity to access natural vitamins from herbage, and vitamin D_3_ from ultraviolet (UV) radiation in sunlight [5–7] in addition to what is provided in their formulated feed. Ranging outside may also reduce feather pecking behaviour to improve plumage coverage and encourage greater expression of natural behaviours such as foraging, walking, and dust bathing [8]. However, hens have a choice in accessing the outdoor area and despite the apparent appeal of an outdoor range, usage can sometimes be low [8] which can result in negative perceptions of the industry by consumers (Pers. comm. to DLMC, 2019). Hen ranging patterns can be affected by many variables such as stocking density [9, 10], flock size, [11, 12] hen age [13], behavioural traits such as fearfulness [14], number of pop-holes [15], rearing environments [11, 16], cover or artificial shelter in the range [17, 18], and weather conditions [19]. The relative contributions of different factors in influencing range use may vary across different flocks housed in different regions [11].

In specific regard to weather factors, temperature has shown a parabolic effect with hens’ range use in the Northern Hemisphere [20, 21]. Wind speed, humidity, and rainfall will also impact ranging with hens preferring milder conditions, but the relative impacts of these variables depend on the season and surrounding temperatures [20–22]. ‘Mild’ may also depend on what conditions hens are accustomed to. For both free-range laying hens and broilers, there are time of day effects with more birds preferring to use the range in the morning and late afternoon/evening and range use varies across seasons [20, 23, 24]. Free-range hens have been shown to increase range use with increasing hours of sunshine, but this effect was only observed at lower temperatures suggesting the sun had a warming effect [21]. In contrast, free-range broilers have been observed to range less when the sun is ‘bright’ versus covered by clouds [24] and will use shade more on sunny days in summer [25]. Quantified solar radiation has also shown a negative relationship with range use with fewer slow-growing broiler chickens ranging as the radiation increased but this relationship was dependent on the type of shelter available on the range [26]. However, while these aforementioned studies have demonstrated relationships between range use and climatic variables, they have all been conducted within European countries which may not be directly applicable to the more extreme sunlight and temperatures experienced in Australia. Furthermore, these previous studies have only observed sunshine or recorded total solar radiation which does not provide an understanding of how specific wavelengths of sunlight affect range usage.

The sun in Australia is extreme due to its geographical position and amount of stratospheric ozone with the north of the country experiencing more extreme sunlight intensity and UV radiation than in Europe [27]. The electromagnetic spectrum containing UV radiation is divided into UVA: 315–400 nm, UVB: 280–315 nm, and UVC: 100–280 nm. Lower UV wavelengths are absorbed by the ozone layer but most radiation from 300–400 nm does reach the earth’s surface [27] where UVB can have damaging effects [28, 29]. The level of UV radiation on the surface depends upon the latitude, season, time of the day, and cloud cover [30]. The UV Index is a numerical value calculated based on a range of factors to quantify risk to human skin health and levels are categorised as low (≤ 2), moderate (3–5), high (6–7), very high (8–10), and extreme (≥ 11) [31]. The UV index and dose values increase from spring (September-November) to summer (December-February) and decrease gradually from autumn (March-May) to winter (June-August), with the maximum values (~15 UV Index and 8 kJ/m^2^ UV dose) observed during the summer in Australia [30]. In clear skies, the annual average UV index across Australia ranges from moderate to extreme from the south to the north of the country respectively, and fluctuates year-round due to the longitudinal variation of the total ozone coverage in the stratosphere [30].

In experimental choice trials, hens show preferences for a lower level of UV exposure and will exhibit different behavioural profiles under full spectrum (includes UVA or both UVA and UVB) versus control lighting [32, 33]. Hens may be motivated to access some UV exposure for the health benefits through stimulation of the vitamin D_3_ pathways [7] but high intensities of UV radiation could result in skin damage [28, 29]. On Australian commercial farms, observations of hen ranging behavioural patterns have documented lower range use across the midday/early afternoon period when the sun is most intense and during the summer months [34, 35]. This may be a consequence of the damaging effect of high UV radiation [28, 29] as hens have shown preferences for range structures that block the greatest amount of UV radiation [34–36], including observations on one of the same farms as reported on in the current study [35]. Alternatively, birds may be avoiding increased brightness (photosynthetically active radiation; PAR) which is the light visible to hens and humans (400–700 nm) and may be visually aversive (similar to humans preferring sunglasses on bright sunny days) or avoiding increased temperatures as a result of sunlight’s infrared radiation (IR) (700 nm- 1 mm). The earth’s surface absorbs heat from the IR of sunlight, which converts into thermal energy increasing surrounding temperatures. In contrast, clouds reduce the amount of incident solar radiation via backscattering and absorption reducing sunlight intensity. In direct sunlight (overhead), the outdoor intensity could be as high as 130,000 lux with average sunny day intensity of 10,000–25,000 lux, up to 1000 lux when overcast, and lowering to 10–40 lux at twilight [37]. To the best of the authors’ knowledge, there are currently no studies that have compared hen ranging patterns outside in relation to the wavelength ranges of sunlight across several months.

The objective of this study was to determine if range use was correlated with sunlight variables across the summer/autumn period on commercial Australian free-range laying hen farms. It was predicted that the hens would show lower use of the range area when the sunlight was most extreme and that different wavelengths of sunlight would have varying impacts on ranging both across the daytime and across months.

## Materials and methods

All the animal protocols and procedures of the study were approved by the Wildlife, Livestock and Laboratory Animal, Animal Ethics Committee of the Commonwealth Scientific and Industrial Research Organisation (Approval numbers: ARA2019/30, ARA2020/27). However, animal husbandry and management fell under the responsibility of the commercial farms.

### Animals and housing conditions

Laying hens (*Gallus gallus domesticus*) housed in commercial free-range systems were used in this study. Three different Australian commercial free-range farms in Tasmania (TAS), Queensland (QLD), and Western Australia (WA) with a diversity of climatic conditions were observed. A total of approximately 70,000 (20,000–30.000/farm) hens were housed and managed according to individual farm protocols, and current standards and guidelines. Studies were conducted across the summer/autumn seasons (December to May) in TAS and QLD in 2019/20 and in WA in 2020/21. On each farm, only a single shed and associated range area was selected for the study. These indoor sheds were furnished with perches, feeders, drinkers, and nest boxes to meet the national poultry guidelines [38] with littered floor and access to an outdoor range area. The lighting, temperature, and ventilation were automatically controlled, however, this varied depending on the sites (see details in the *Study Sites* sections).

### Study sites

#### Tasmania

**Fig 1:**
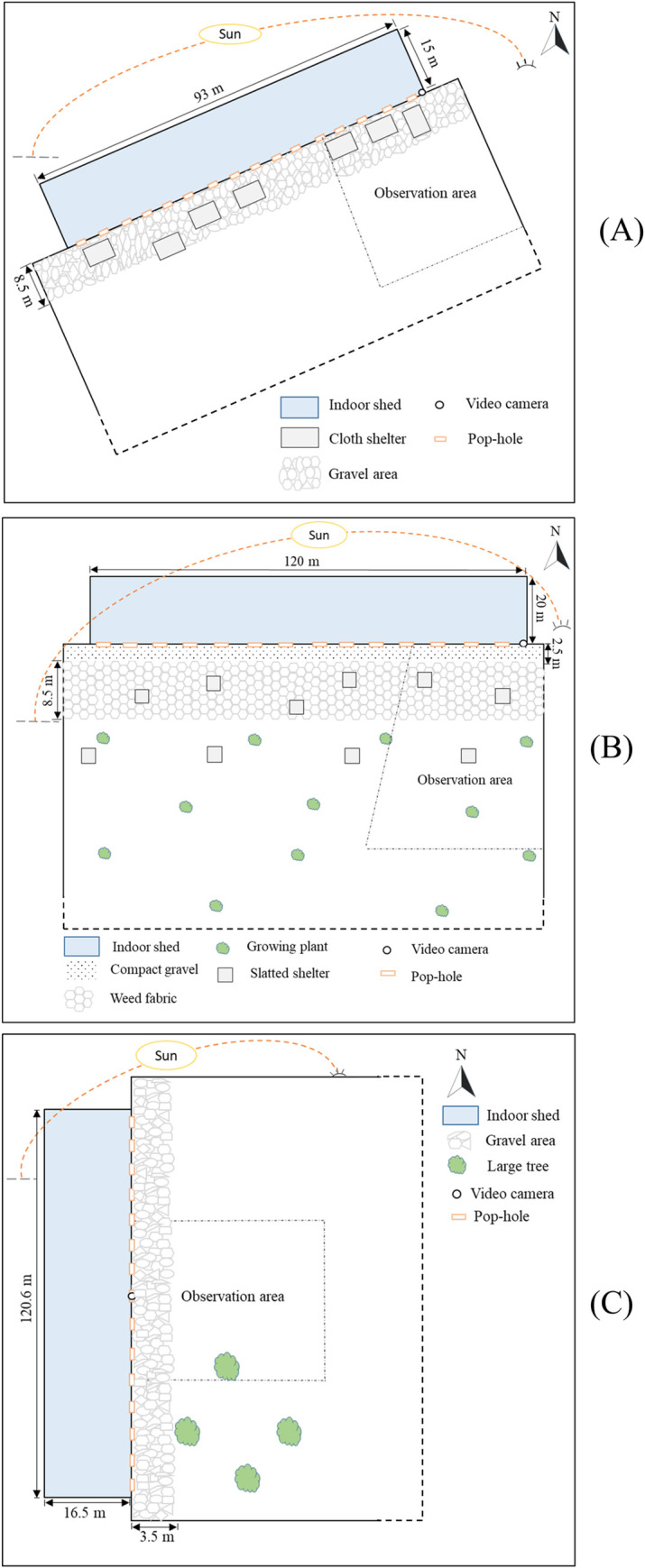
Schematic diagram of the study farms: TAS (A), QLD (B), and WA (C) showing the indoor shed and range areas including shelters on the range, video camera placement, observation area, and pathway of the sun across the day. Diagrammatic representation only and not drawn to scale where dashed perimeter lines indicate the ranges extended beyond what is drawn.

Tasmania (TAS) is the most southern island state of Australia with relatively cooler temperatures and lower UV indices compared to other states of the country [27]. The study farm was located in the northern midlands of Tasmania. An estimated 20,000 Hy-Line Brown laying hens of 20 weeks of age in one flock were studied across the summer/autumn months (21 December 2019 to 31 March 2020). The day-old chicks were reared indoors until transfer at 14 weeks to the free-range indoor shed and housed with standard management practices as per the Model Code of Practice for Domestic Poultry [38]. The indoor shed (93 m L x 15 m W x 3.5 m H) was in the northeast-southwest direction and contained an aviary system with a stocking density of 14 hens/m^2^ (Fig 1A). The sidewalls of the shed were made of cool room panels from the ground to 0.7 m height with the remainder covered with automatic curtains. Feed and water were provided *ad libitum* inside the shed only. Adjacent to the sidewalls was an outdoor range area on both the north and south sides with a maximum outdoor stocking density of 6,666 hens/ha (equivalent to 0.67 hens/m^2^). However, only the south side of the shed was studied as farm management personnel indicated more birds ranged on that side. Hens could access the range area through pop-holes from 20 weeks of age. Sixteen pop-holes (0.9 m L x 0.6 m H) were set at 10 cm above the ground on the south side and were regulated automatically but the opening time varied based on the day lengths (opened at 11:00 and 10:30 during 21 December—21 February and 22 February—31 March, respectively) and closed at 21:00. Most of the range area was covered with perennial ryegrass, clover, and native pasture starting at 15 m out from the shed wall. Adjacent to the shed wall was 8.5 m of unevenly distributed stones of varying size followed by a sloping area approximately 7 m wide. There was no visible degradation of the grass in the range area during the study period. No trees were present on the range but seven rectangular shade cloth covers (6.5 m L x 4.6 m W x 1 m H) were set within the gravel area at least 1 m from the sidewall of the shed (Fig 1A). One shade cover from each corner was shifted farther away (approx. 18 m) from the shed on 03 March (commercial decision, unrelated to the project aims) and remained there until the completion of the study. The boundaries of the range area were wire fences.

#### Queensland

Queensland (QLD) is the north-eastern state that experiences the highest average maximum temperature and second-highest UV indices (after the Northern Territory) in Australia [27]. The selected commercial free-range laying hen farm for this study was situated in the south-west part of the Queensland state. The study was conducted within a flock of approximately 20,000 Hy-Line Brown laying hens across the summer/autumn months (23 December 2019 to 16 April 2020). In this commercial set-up, pullets were reared indoors until transfer at 16 weeks of age to the free-range indoor shed where they were managed as per the national guidelines within the Model Code of Practice for Domestic Poultry [38]. The indoor shed (120 m L x 20 m W x 8 m H) contained an aviary system and was long in the east-west position with an indoor stocking density of 9 hens/m^2^ (Fig 1B). *Ad libitum* feed and water were only provided inside the shed. The sidewalls were made of solid materials (poly panel) from the ground up to 0.55 m height and curtains covered the remainder of the wall up to the ceiling. The shed had outdoor ranges on both the north and south sides with an approximate stocking density of 1500 hens/ha (equivalent to 0.15 hens/m^2^). Hens within the shed could only access the range on either the north or south face due to an internal shed division, thus each shed actually contained 40,000 hens total. The south side of the shed was used for this study. Hens could access the outdoor range at 20 weeks of age via pop-holes. In the south side, the sidewall of the shed had 14 pop-holes (6 m L x 0.6 m H) at 5 cm above the ground, but typically only half were opened across the shed length in an alternating pattern during the study period. The automatic pop-holes were opened at 09:00 and closed at 20:00 daily; however, during adverse weather conditions such as extreme heat or storms they remained closed to ensure the safety of the hens. The range area adjacent to the shed wall (2.5 m) was covered with evenly distributed compact gravel, followed by 12 m of range area covered with heavy weed fabric, then an approximately 52 m distance was uncovered (dirt), and the remainder of the range area was covered with grass (Fig 1B). A number of growing trees planted 8 m apart were present within the dirt area in four parallel rows starting at 6.5 m from the weed fabric out into the range. There was visible degradation in the grassed area at the beginning of the study which gradually regrew during the study period. Distributed across the dirt range area there were ten slatted wooden pallet shelters (1.2 m L x 2.2 m W). The boundaries of the range area were wire fences.

#### Western Australia

Western Australia (WA) is the largest state of Australia having a maximum number of sunny days with a clear blue sky year-round. The farm selected for this study was located in the Wheatbelt region of Western Australia that is characterised by its hot dry summers and mild winters. A flock of approximately 30,000 Lohmann Brown hens within one shed on the farm was studied across the summer/autumn months (28 January 2020 to 20 May 2021). Hens were transferred from a commercial rearing facility into the indoor shed (120.6 m L x 16.5 m W x 4.83 m H) of the free-range farm at 15 weeks of age (Fig 1C). The housing practices followed the national guidelines within the Model Code of Practice for Domestic Poultry [38]. The indoor shed was in a north-south direction and contained an aviary system. Feed and water were provided *ad libitum* inside the shed only. The base of the shed sidewalls (1 m) was made from sandwich panel and the remainder of the walls were covered by curtains up to the ceiling. Hens could access the outdoor range from 19 weeks of age via pop-holes on both sides of the shed but only the east side of the shed was used for this study. The indoor and outdoor stocking density of hens was 10.75 hens/m^2^ and 1500 hens/ha (equivalent to 0.15 hens/m^2^), respectively. Pop-holes for range access were located 5 cm above ground in the sidewall. There were 15 pop-holes (2.06 m L x 0.35 m H) in the south side through which hens had access to the range from 09:00 to 19:00. However, the pop-holes remained closed preventing range access during extreme temperatures of 38°C or above. The range area adjacent to the shed wall (3.5 m) was covered with unevenly distributed gravel, then the immediate range area of approximately 10 m was uncovered (dirt), followed by approximately 12.5 m of range area covered with perennial rye pasture (Fig 1C). A further approximately 40 m of range area had a mix of bottle brush trees (*Callistemon* spp.) and dirt as well. There were a few large trees (*Eucalyptus* spp.) grown at the southeast corner of the range area. The boundaries of the range area were wire fences.

### On-farm weather stations

An MEA weather station (Green Brain, 41 Vine Street, Magill, SA 5072, Australia) was set-up on each farm site for recording sunlight variables and weather data every 15 min over the study periods. The weather station was mounted on a post (user supplied, 1 m height as recommended by the commercial provider) with different sensors (UV3pAB UV pyranometer, QS5 PAR pyranometer, and SR05-D1A3 pyranometer) for recording of sunlight variables including UV radiation (UV_AB_) (288–432 nm) (W/m^2^), PAR (400–700 nm) (μmol/m^2^/s), and total solar radiation (TSR) (285 nm-3000 nm) (W/m^2^), respectively. The TSR included UV_AB_ wavelengths, PAR and IR and was used to extract IR (700 nm-3000 nm) (W/m^2^). Additionally, an air temperature and relative humidity sensor recorded weather variables including ambient temperature (˚C), relative humidity (%), barometric pressure (mBar), dew point (˚C), and vapour pressure deficit (kPa). As the study objective was to establish the relationship between sunlight variables and hen ranging behavioural patterns, only the solar radiation spectrums, ambient temperature, and relative humidity weather data were considered in the final analyses.

### Video recording

A high-resolution Hikvision (Hangzhou, Zhejiang, China) security camera system (Hikvision DS-7608NI-I2/8P CCTV NVR Recorder and Hikvision DS-2CD2355FWD-I CCTV 6MP Turret cameras) was installed at the southeast corner in both TAS and QLD, and at the middle of the indoor shed just above the central pop-hole in WA to record hen range use across the study periods. Thus, we recorded part of the southeast corner of the range area for TAS (Fig 1A) and QLD (Fig 1B), and part of the middle portion of the range area for WA (Fig 1C). It was not logistically feasible to record the range areas in entirety given their large size. The area to be video recorded was first discussed with the managers of the commercial farms to ensure a representative area was selected based on typical ranging patterns for the flock of interest. The video recording was continuous daily during range access time across each study period.

### Data collection

For observation of hen ranging behavioural patterns, the number of hens using the outdoor range in the sampled area for each farm was recorded daily across the study periods. A single observer took image snapshots at 30 min intervals on all available days during the period of ranging time, from pop-hole opening until sunset to count the number of hens ranging outside. The initial image snapshot was taken just 3 min after pop-hole opening with all other snapshots taken on the hour/half hour (i.e., if the pop-hole opened at 09:00, the first snapshot was taken at 09:03, the next snapshot at 09:30, 10:00 and so forth until sunset). Observations indicated hens rapidly accessed the range area within approximately two minutes of the pop-holes opening. The exact area of the range that was sampled for counting hens varied among the farms based on hen visibility through the video camera and their typical range occupancy (i.e., some areas within the image snapshot were rarely accessed by hens) but consistency in observational area was maintained within each farm over the study periods (Fig 1A–1C). During sunny time points (i.e., sun was not obscured by clouds), hen counts were categorised as the number of hens under the direct sunlight (‘sun’), and the number of hens in the clearly visible shaded areas from range shelters or the shed (‘sun-shade’). However, in cloudy conditions (i.e., image snapshots were duller) where shade was not visibly distinct, the same parts of the range area were marked from the previous sunny day and categorised as the number of hens under the cloud (‘cloud’), and the number of hens in the shaded areas from range shelters or the shed (‘cloud-shade’). Moreover, in the late afternoon/evening when the demarcation line between the sun and shade was not visibly distinct, the counts of hens were only considered as being in the ‘sun-shade’ and these data were later excluded from the statistical analyses. Hens were counted using the Image-J 1.53a software (Wayne Rasband, National Institute of Health, MA, USA) individually within the specified range area. However, when piling occurred (i.e., hens formed dense groups) and individual hens were not all clearly identifiable, the number of hens was estimated in the group by counting the hens within a certain area and then estimating the total count by multiplying the counted area. This same guideline was followed in future similar occurrences. Hens within the shade and under the shelter that were clearly identifiable on the image snapshot were counted but any hens directly under the range shelter were unable to be taken into account. TAS had a total of 101 days available data of hens ranging outside; while QLD and WA had a total of 94 days and 67 days, respectively. However, the days were not consecutive because of faults in the recording system as well as pop-hole closures during adverse weather conditions. As the hen observational data were collected at 30 min intervals, the corresponding weather parameters across the 15-min period directly prior to the observation time point were matched accordingly. Due to the distinctness of each farm, databases were prepared and analysed separately.

### Statistical analyses

This study generated data on the number of hens on the range across the day and this was correlated with recorded weather parameters on the farms. Only one shed was observed per farm and analysed separately due to the discrepancies in the farms’ structures and climatic conditions. For TAS, a total of 101 days of hen counts at 30 min intervals from pop-hole opening (10:30/11:00) until sunset (21:00) were analysed. For the analyses by month, data were grouped into December (11 days), January (31 days), February (29 days), and March (30 days). For QLD, a total of 94 days of hen counts at 30 min intervals from pop-hole opening (09:00) until sunset (20:00) were analysed. For the analyses by month, data were grouped into December/January (27 days), February (26 days), March (29 days), and April (12 days). For WA, a total of 67 days of hen counts at 30 min intervals from pop-hole opening (09:00) until sunset (19:00) were analysed. For the analyses by month, data were grouped into January/February (9 days), March (14 days), April (26 days), and May (18 days). The combined adjacent months for QLD and WA were due to low numbers of available observation days.

The principal response variables were the number of hens under the direct sunlight (‘sun’) and ‘sun-shade’ and the number of hens in ‘cloud’ and ‘cloud-shade’ across the day from pop-hole opening until sunset during the sunny and cloudy conditions, respectively. Both ‘sun/sun-shade’ and ‘cloud/cloud-shade’ data were analysed individually (12 separate datasets: 3 farms x 4 environments of ‘sun’/’sun-shade’ and ‘cloud’/’cloud-shade’). The independent variables were different levels of sunlight spectrums including UV_AB_, PAR, and IR, and the weather variables including ambient temperature and relative humidity. TSR readings were only used for extracting IR by subtracting the UV_AB_ and PAR. An approximation conversion value (μmol/m^2^/s to W/m^2^) as described by Thimijan and Heins [39] was applied to the PAR readings so all measures were in the same units for calculating the IR values. Given the conversion is an approximation, there was a margin of error in the IR calculation in the evening hours or during heavy rain. There were 23 data points (out of 4481) that were calculated as negative, and those data were excluded from the final dataset. The hen count data of ‘sun’ and ‘cloud’ were log (x+1) transformed to approach data normality as well as to include ‘0’ values (when no hens were present) and the hen counts for ‘sun-shade’ and ‘cloud-shade’ data required square-root transformation. The sunlight and weather data met the requirements of parametric statistics, so no transformations were required. Using JMP^®^ 16.0 (SAS Institute, Cary, NC, USA), general linear models (GLM) were applied with α level set at 0.05 on the number of hens in the ‘sun’ and ‘cloud’ separately to determine if the ‘time of day’ and ‘month’ as fixed factors had influence on the hens’ range distribution in unshaded range areas during sunny and cloudy conditions, respectively. An interaction between the fixed factors was not included as the counts across time of day were not balanced across months resulting from changes in day length and pop-hole opening times. Separate models with the same parameters were also fitted to assess hens’ presence in the ‘sun-shade’ and ‘cloud-shade’ areas of the range. The studentised model residuals were visually inspected for confirming homoscedasticity. Where significant differences were present, post hoc Student’s t-tests were applied to the least squares means with Bonferroni corrections to the α level to account for more than 4 post-hoc comparisons. The weather and sunlight spectrum data were compiled and the mean differences among the weather variables and sunlight spectral intensities presented between the months.

To assess the effects of sunlight and climatic conditions on range use across the entire study period, multiple linear regression analyses with sunlight (UV_AB_, PAR, and IR) and weather (ambient temperature and relative humidity) variables as predictors were performed with the number of hens in ‘sun’ and ‘cloud’ separately for individual farms. However, before running the model, the collinearity among the independent variables were checked through determination of variance inflation factors (VIF). Because of the collinearity effect (VIF ≥ 10) among the sunlight variables, ridge regression analyses were chosen [40] to best fit the predictors into the model. Therefore, we used the *‘lmridge’* package in R statistical software [41] for the ridge regression. While this regression is recommended for multicollinearity, it was difficult to control for all collinearities given the direct relationship between different sunlight wavelengths. The relative weight between the independent predictors in the regression model were estimated by the R package *‘relaimpo’* [42]. Initially, all independent variables were fitted into the model, then the non-significant variables (p > 0.10) were removed through backward stepwise elimination to reach the model of best fit based on the adjusted-R^2^ values. To determine how sunlight and weather variables may affect the hens’ use of the range under direct sunlight (‘sun’) across the months in different climatic conditions, separate ridge regression models were also performed for each month(s) with the number of hens in the ‘sun’ included as the dependent variable, and the sunlight (UV_AB_, PAR, and IR), and weather (ambient temperature and relative humidity) variables included as independent variables. Through backward stepwise elimination any non-significant variables (p > 0.10*)* were removed to reach the model of best fit based on the adjusted-R^2^ values. Similar ridge regression models were also applied for the number of hens in the ‘sun-shade’ and ‘cloud-shade’ to assess the relationship with the sunlight and weather parameters across the entire study period. Individual ridge regression models by month(s) for hens in the ‘cloud’ were not performed as a representative sample of cloudy conditions was not available for each month. Additionally, individual ridge models by month(s) for either ‘sun-shade’ and ‘cloud-shade’ were not applied as the weather stations were set-up to be exposed to direct sunlight and thus there were no measurements of the specific environmental conditions within shaded areas on the range, limiting interpretations. Only raw values are presented in the figures.

## Results

### Tasmania (TAS)

Due to the farm layout, the shade created by the indoor shed and range shelters increased steadily into the range across the day. The shade was greatest at the end of the day at approximately 9.5 m and 12 m (at the beginning and end of the study period, respectively) from the indoor shed sidewalls in the sunlight (when the demarcation line between the ‘sun’ and ‘shade’ portions were distinct). Although the ‘shade’ area increased across the day, there was still ample range area available in full sunlight.

#### Weather conditions and sunlight intensity

During the study period, the average ambient temperature and relative humidity was 21.2 ± 0.10˚C (ranged from 9.8 to 36.7˚C) and 50.3 ± 0.39% (ranged from 16.7 to 98.4%), respectively. The average sunlight irradiance was recorded as UV_AB_ irradiance 3.6 ± 0.06 W/m^2^ (ranged from 0.1 to 9.2 W/m^2^), PAR irradiance 1122.2 ± 16.15 μmol/m^2^/s (ranged from 6 to 2536 μmol/m^2^/s), and IR irradiance 258.4 ± 4.00 W/m^2^ (ranged from 0.06 to 606.9 W/m^2^). Both weather conditions and sunlight irradiance varied across the months as expected (Table 1).

**Table 1:** Mean daily (± SEM) outdoor conditions during ranging time across the months in TAS (n = 101 days).

Variables^1^	December	January	February	March
Ambient temperature (˚C)	25.2 ± 0.27	22.7 ± 0.15	20.7 ± 0.16	18.4 ± 0.17
Relative humidity (%)	33.2 ± 1.03	45.8 ± 0.60	51.8 ± 0.63	60.6 ± 0.65
UV_AB_ (W/m^2^)	4.7 ± 0.17	3.8 ± 0.10	3.6 ± 0.11	2.9 ± 0.11
PAR (μmol/m^2^/s)	1533.9 ± 47.49	1164.5 ± 27.53	1115.6 ± 29.03	917.6 ± 29.79
IR (W/m^2^)	340.4 ± 11.87	266.4 ± 6.88	256.6 ± 7.25	218.5 ± 7.44

^1^UV_AB_ (ultraviolet radiation A and B wavelengths), PAR (photosynthetically active radiation), IR (infrared radiation).

#### Effects of time of day on hens’ distribution outside

**Fig 2:**
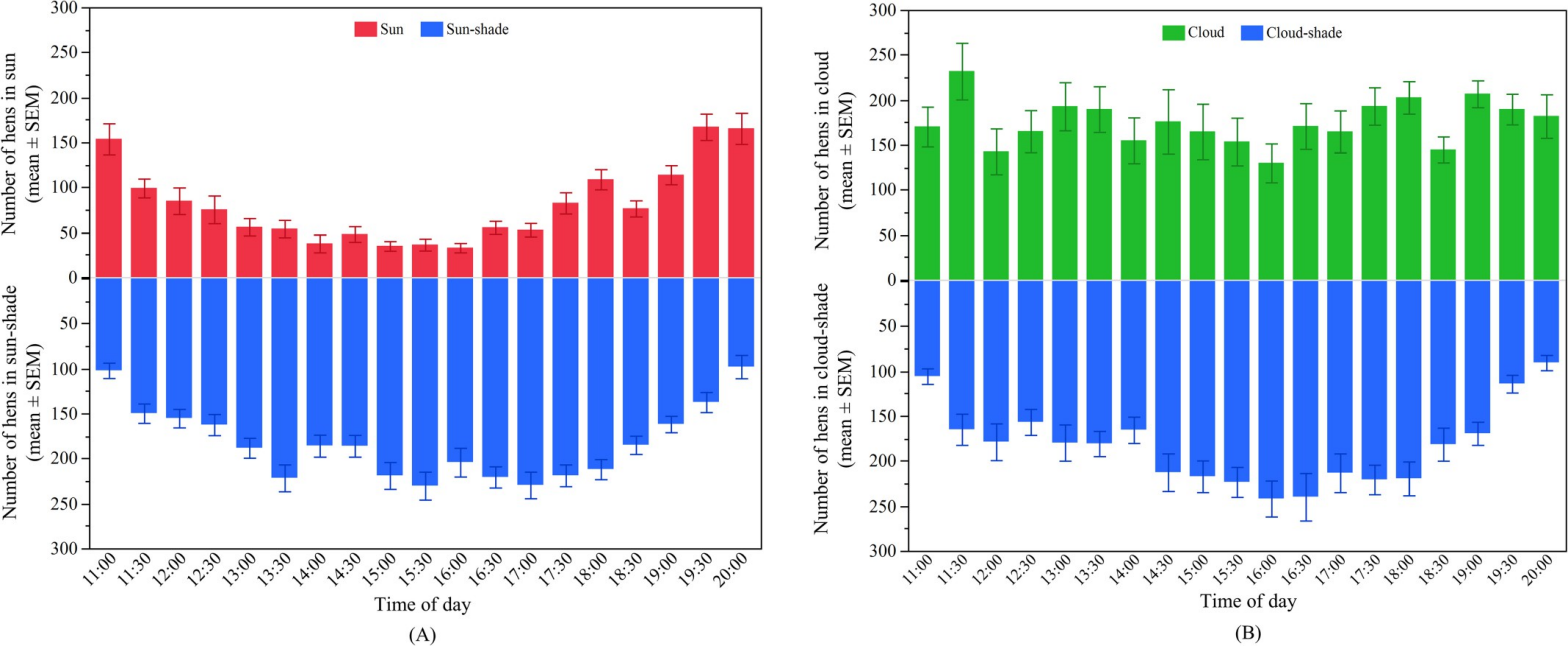
Distribution of hens on the range across the day in TAS: (A) The mean (± SEM) number of hens in the ‘sun’ (top) and ‘sun-shade’ (bottom); (B) The mean (± SEM) number of hens in the ‘cloud’ (top) and ‘cloud-shade’ (bottom).

The number of hens in the ‘sun’ significantly varied across the daytime (F_18, 1147_ = 31.79, p < 0.0001; Fig 2) with peaks in the morning and evening (p < 0.003). The number of hens in the ‘sun-shade’ was also affected by time of day (F_18, 1147_ = 11.55, p < 0.0001; Fig 2) but with an opposite pattern to that observed for hens in the ‘sun’ with the lowest values observed in the morning and evening (p < 0.003).

The overall number of hens in the ‘cloud’ also significantly varied across the daytime (F_18, 600_ = 1.93, p = 0.01; Fig 2). However, across most of the time points, similar numbers of hens were observed except for a slight increase between 19:00 and 19:30 and a decrease between 15:30 and 16:00 compared to the rest of the day (p < 0.003). The number of hens in the ‘cloud-shade’ was also affected by time of day (F_18, 600_ = 7.54, p < 0.0001; Fig 2) with a greater number of hens observed during the midday and afternoon periods (p < 0.003).

#### Differences in hens’ distribution across months

**Fig 3:**
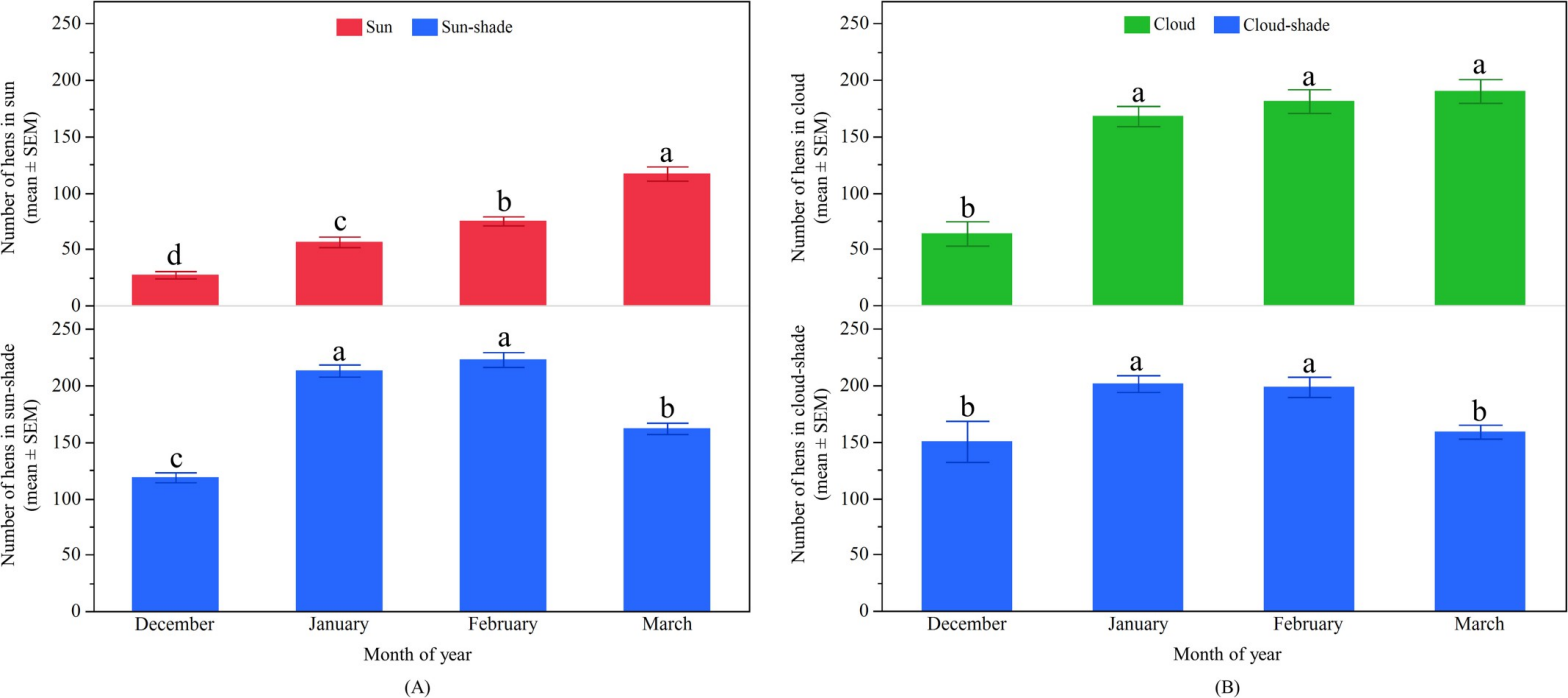
Distribution of hens on the range across months in TAS: (A) The mean (± SEM) number of hens in the ‘sun’ (top) and ‘sun-shade’ (bottom); (B) The mean (± SEM) number of hens in the ‘cloud’ (top) and ‘cloud-shade’ (bottom). ^a-d^ Dissimilar superscript letters indicate significant differences among months. Raw values are presented with analyses conducted on transformed data.

There was a significant effect of month on the number of hens observed in the ‘sun’ (F_3, 1147_ = 202.02, p < 0.0001; Fig 3). The greatest number of hens ranged in the ‘sun’ during the month of March, followed by February, January, and then December. The number of hens in the ‘sun-shade’ was also significantly influenced by month of observation with a peak in January and February, and the fewest hens in December (F_3, 1147_ = 74.00, p < 0.0001; Fig 3). There was a significant effect of month on the number of hens in the ‘cloud’ (F_3, 600_ = 6.83, p < 0.001) with the fewest hens observed in December (Fig 3). Similarly, the number of hens ranging in the ‘cloud-shade’ varied across the months (F_3, 600_ = 13.48, p < 0.0001) with the most hens observed in January and February (Fig 3).

#### Relationship between sunlight and hens’ range use

**Fig 4:**
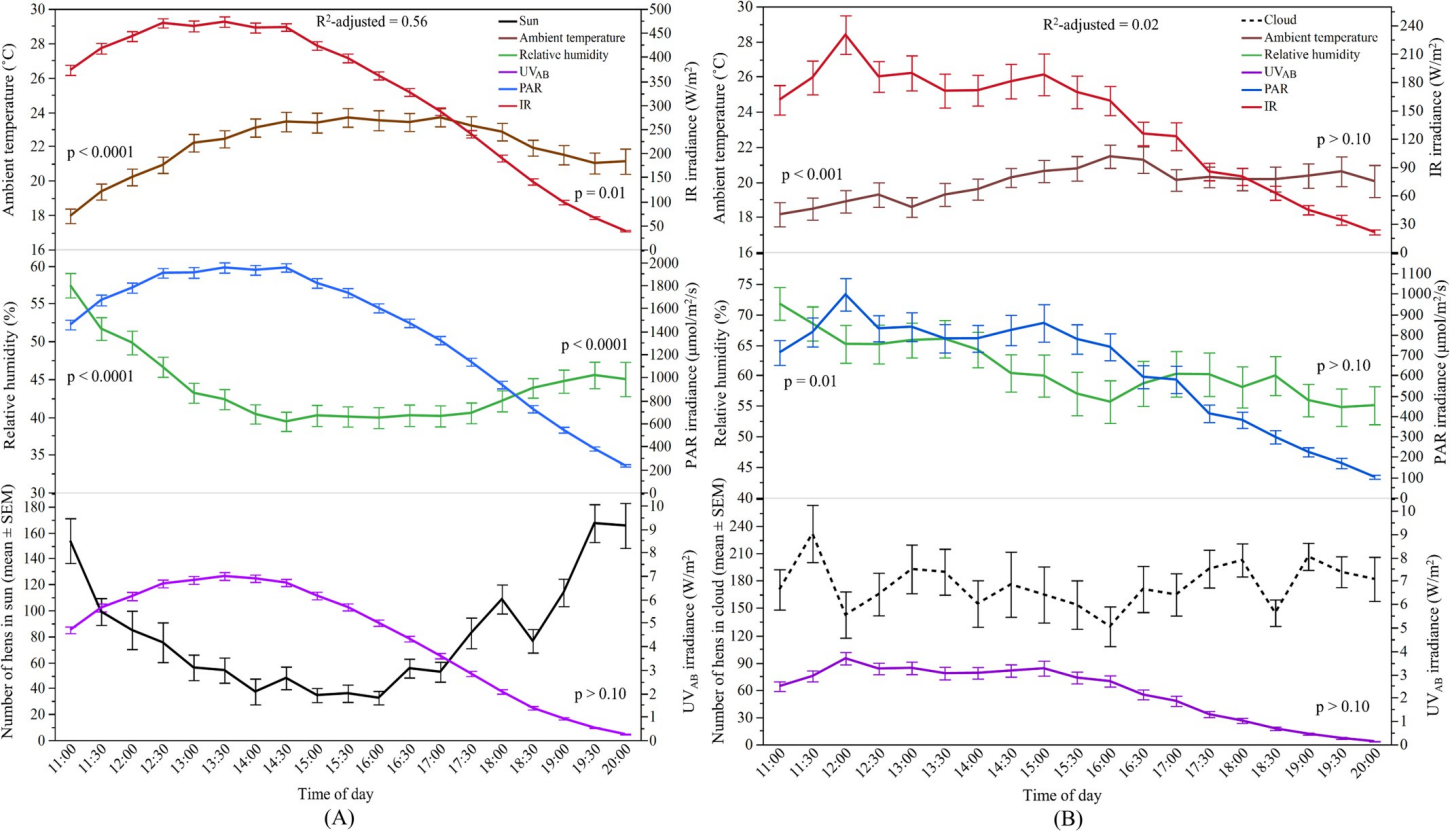
Relationship between sunlight spectrums and weather variables with the number of hens in the ‘sun’ and ‘cloud’ in TAS: (A) Y-axis (left): The mean (±SEM) number of hens in the ‘sun’, and the mean (±SEM) relative humidity and ambient temperature; Y-axis (right): the mean (±SEM) irradiance of UV_AB_ (ultraviolet radiation A and B wavelengths), PAR (photosynthetically active radiation), and IR (infrared radiation); (B) Y-axis (left): The mean (±SEM) number of hens in the ‘cloud’, and the mean (±SEM) relative humidity and ambient temperature; Y-axis (right): the mean (±SEM) irradiance of UV_AB_ (ultraviolet radiation A and B wavelengths), PAR (photosynthetically active radiation), and IR (infrared radiation). p > 0.10 indicates the variable had no significant effect and was removed from the final model.

The overall ridge regression model to determine the effects of sunlight and weather variables on the number of hens in the ‘sun’ across the entire study period was significant (F_3.18, 1165.56_ = 410.20, p < 0.0001, Fig 4). The model showed that the number of hens in the ‘sun’ could be predicted significantly by ambient temperature, relative humidity, PAR and IR, and all these factors explained 56% of the variance. Both ambient temperature and PAR were strongly negatively correlated and contributed to the model 33.8% and 24.8%, respectively showing that increased air temperature and PAR irradiance resulted in fewer hens ranging in the ‘sun’. Relative humidity and IR had a positive relationship with the number of hens in the ‘sun’ and caused 22.9% and 18.5% of the models’ variation, respectively. When an overall model for the number of hens in the ‘cloud’ was run, only weather variables contributed to the model (F_1.95, 620_ = 7.22, p = 0.001) showing a negative relationship but explaining only 2% of the variance (Fig 4).

The results of multiple ridge regression models to establish the relationship between the predictors and hens’ distribution in the ‘sun’ per month(s) are presented in Table 2. The maximum variance in the models accounted for by the sunlight and weather variables for the number of hens in the ‘sun’ was found in December (60%). Air temperature, UV_AB_, and PAR showed negative relationships and IR had a positive relationship (Table 2). The effects of sunlight and weather variables in the models gradually decreased until the least variance was explained in March (18%). Ambient temperature and PAR significantly contributed to the models of January and February, and both had negative relationships with the number of hens in the ‘sun’. However, sunlight variables (UV_AB_, and IR) did not have an effect in the individual model for March indicating seasonal variation in factors affecting hen ranging.

**Table 2:** Multiple ridge regression analyses (ridge parameter, k = 0.02) on the number of hens in the ‘sun’ across the day in TAS for different observation months. Only variables that significantly contributed to the most parsimonious model are presented.

Month	Predictor^1^	β-coefficient (Standardised)^ǂ^	t-value	P-value	Model’s F-statistics	Relative weight of the predictors in the model
December	Ambient temperature	-0.52	-11.41	< 0.0001	R^2^-adjusted = 0.60 F_2.46, 173.17_ = 84.98, p < 0.0001	37.3%
	UV_AB_	-0.21	-1.77	< 0.0001		14.9%
	PAR	-0.68	-5.84	0.08		27.9%
	IR	0.41	4.25	< 0.0001		20.0%
January	Ambient temperature	-0.42	-10.66	< 0.0001	R^2^-adjusted = 0.58 F_3.20, 343.54_ = 135.53, p < 0.0001	34.3%
	Relative humidity	0.20	5.05	< 0.0001		20.9%
	PAR	-0.55	-7.15	< 0.0001		25.0%
	IR	0.14	1.83	0.07		19.9%
February	Ambient temperature	-0.32	-7.00	< 0.0001	R^2^-adjusted = 0.29 F_2.94, 330_ = 47.06, p < 0.0001	39.5%
	Relative humidity	0.15	3.17	0.002		11.4%
	PAR	-0.35	-7.58	<0.0001		49.1%
March	Ambient temperature	-0.09	-1.62	0.10	R^2^-adjusted = 0.18 F_2.93, 310_ = 24.46, p < 0.0001	12.1%
	Relative humidity	0.28	5.17	< 0.0001		50.4%
	PAR	-0.25	-4.97	< 0.0001		37.6%

^ǂ^β-coefficients (standardised) of the predictor variables were estimated separately using the ridge regression coefficient in ‘R’ as the original ridge package did not include the ‘β-coefficient’ value in the regression outputs. ^1^UV_AB_ (ultraviolet radiation A and B wavelengths), PAR (photosynthetically active radiation), IR (infrared radiation).

Overall models for the number of hens in the ‘sun-shade’ and ‘cloud-shade’ were run separately and the results are presented in Table 3. The model for the number of hens in the ‘sun-shade’ showed that ambient temperature, relative humidity, and PAR had significant positive relationships while IR had a negative relationship. Nevertheless, the model explained only 2% of the variance. The model of hens in the ‘cloud-shade’ accounted for 8% of the variance with a positive trend for relative humidity, and a positive strong relationship for ambient temperature and UV_AB_ suggesting an increase in ambient temperature and UV_AB_ resulted in more hens being in the shaded areas during cloudy conditions.

**Table 3:** Multiple ridge regression analyses (ridge parameter, k = 0.02) on the number of hens in the ‘sun-shade’ and ‘cloud-shade’ across the day in TAS for all observation months. Only variables that significantly contributed to the most parsimonious model are presented.

Range distribution	Predictor^1^	β-coefficient (Standardised)^ǂ^	t-value	P-value	Model’s F-statistics	Relative weight of the predictors in the model
Sun-shade	Ambient temperature	0.15	4.27	< 0.0001	R^2^-adjusted = 0.02 F_3.18,1165.56_ = 14.18, p < 0.0001	20.3%
	Relative humidity	0.17	5.04	< 0.0001		32.4%
	PAR	0.33	5.09	< 0.0001		25.0%
	IR	-0.28	-4.48	< 0.0001		22.3%
Cloud-shade	Ambient temperature	0.23	5.15	< 0.0001	R^2^-adjusted = 0.08 F_2.92, 619_ = 19.59, p < 0.0001	46.3%
	Relative humidity	0.08	1.83	0.07		6.5%
	UV_AB_	0.21	5.23	<0.0001		47.3%

^ǂ^β-coefficients (standardised) of the predictor variables were estimated separately using the ridge regression coefficient in ‘R’ as the original ridge package did not include the ‘β-coefficient’ value in the regression outputs.

^1^UV_AB_ (ultraviolet radiation A and B wavelengths), PAR (photosynthetically active radiation), IR (infrared radiation).

### Queensland (QLD)

This farm had more plants growing within the range area, but the shade created by these plants was minimal. The indoor shed cast shade onto the range, which increased in size over the study period but reduced in size across the day. Thus, at the start of the observations, the maximum shade measured approximately 2 m in width and increased up to 5.5 m at 09:00 by the end of the study period. There was no shade cast after 15:30 on the first day of observations but it increased over time, and on the last day it measured 3 m at 17:00. The majority of the range area experienced full sun.

#### Weather conditions and sunlight intensity

During the study period, the mean temperature and relative humidity was 27.2 ± 0.10˚C (ranged from 18.4 to 37.2˚C) and 53.4 ± 0.43% (ranged from 16.5 to 99.4%), respectively. The average sunlight irradiance was recorded as UV_AB_ irradiance 4.6 ± 0.07 W/m^2^ (ranged from 0.1 to 11.5 W/m^2^), PAR irradiance 1180.1 ± 17.00 μmol/m^2^/s (ranged from 6 to 2732 μmol/m^2^/s), and IR irradiance 268.6 ± 4.10 W/m^2^ (ranged from 0.06 to 642.7 W/m^2^). Both weather conditions and sunlight irradiance varied across the months as expected (Table 4).

**Table 4:** Mean daily (± SEM) outdoor conditions during ranging time across the months in QLD (n = 94 days).

Variables^1^	December/ January	February	March	April
Ambient temperature (˚C)	30.2 ± 0.15	26.4 ± 0.16	25.8 ± 0.15	25.3 ± 0.24
Relative humidity (%)	43.7 ± 0.69	65.5 ± 0.71	54.6 ± 0.69	45.9 ± 1.10
UV_AB_ (W/m^2^)	5.0 ± 0.13	4.5 ± 0.13	4.6 ± 0.13	4.1 ± 0.20
PAR (μmol/m^2^/s)	1339.3 ± 30.70	1086.5 ± 31.70	1153.2 ± 30.5	1068.7 ± 48.70
IR (W/m^2^)	295.1 ± 7.45	247.3 ± 7.69	274.6 ± 7.42	236.7 ± 11.81

^1^UV_AB_ (ultraviolet radiation A and B wavelengths), PAR (photosynthetically active radiation), IR (infrared radiation).

#### Effects of time of day on hens’ distribution outside

**Fig 5:**
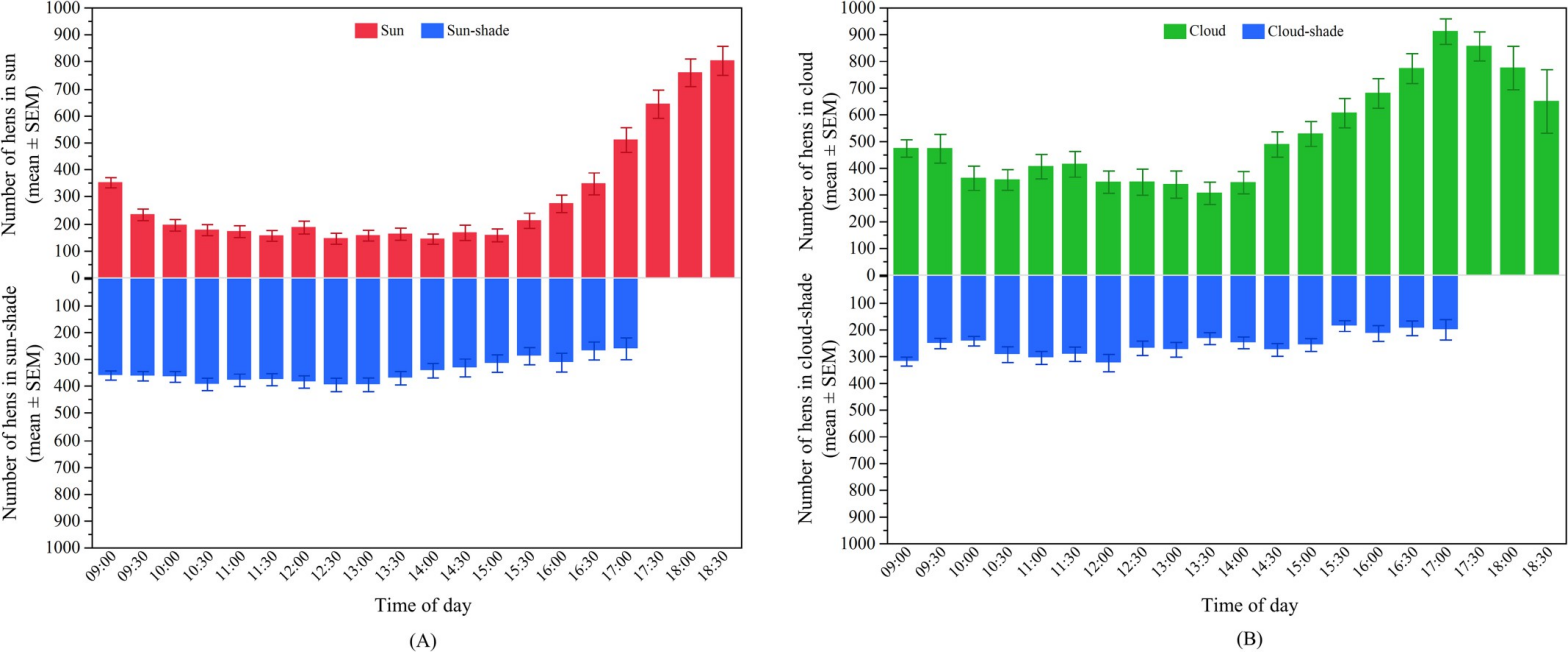
Distribution of hens on the range across the day in QLD: (A) The mean (± SEM) number of hens in the ‘sun’ (top) and ‘sun-shade’ (bottom); (B) The mean (± SEM) number of hens in the ‘cloud’ (top) and ‘cloud-shade’ (bottom).

There were significant effects of time of day on the number of hens in the ‘sun’ (F_19, 1022_ = 26.23, p < 0.0001; Fig 5). Hens had similar preferences for ranging in the ‘sun’ between 10:00 and 15:30, which then gradually increased after 16:00 until the evening (p < 0.003). The number of hens in the ‘sun-shade’ also varied across the day (F_16, 878_ = 9.26, p < 0.0001) in an opposite pattern to the hens in the ‘sun’, however with less variation at most of the time points (p > 0.003; Fig 5).

Time of day also had an effect on the number of hens in the ‘cloud’ (F_19, 674_ = 7.20, p < 0.0001; Fig 5) with similar preferences for ranging in the ‘cloud’ between 10:00 and 14:00. More hens were observed in the morning (between 09:00 and 09:30) compared to midday but the number gradually increased in the afternoon (from 14:30 to 17:00) (p < 0.003). The number of hens in the ‘cloud-shade’ also statistically varied (F_16, 539_ = 7.02, p < 0.0001) but similar patterns were observed across most time points (p > 0.003; Fig 5).

#### Differences in hens’ distribution across months

**Fig 6:**
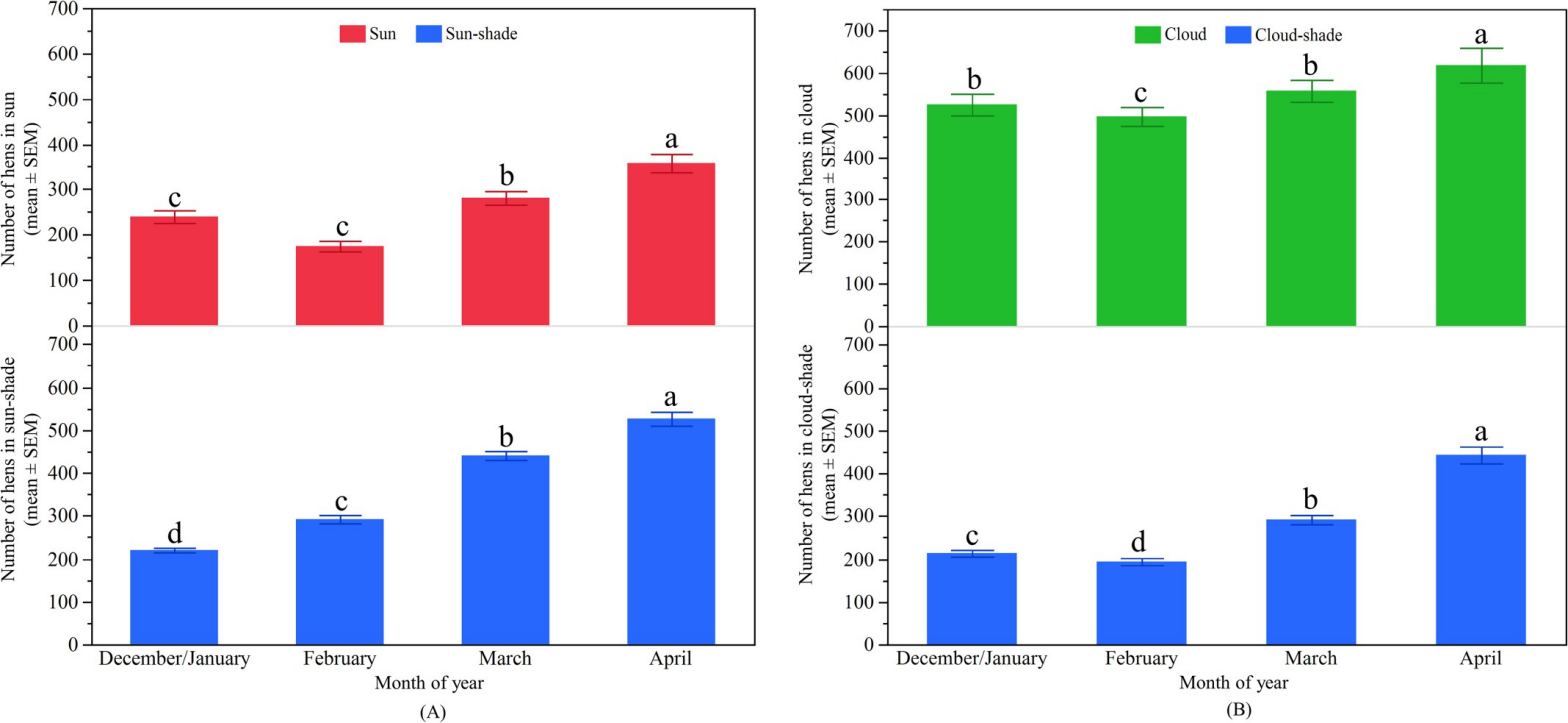
Distribution of hens on the range across months in QLD: (A) The mean (± SEM) number of hens in the ‘sun’ (top) and ‘sun-shade’ (bottom); (B) The mean (± SEM) number of hens in the ‘cloud’ (top) and ‘cloud-shade’ (bottom). ^a-d^ Dissimilar superscript letters indicate significant differences among months. Raw values are presented with analyses conducted on transformed data.

The distribution of hens on the range in the ‘sun’ significantly varied across the months (F_3, 1022_ = 57.27, p < 0.0001; Fig 6). The most hens were observed in the ‘sun’ during April, and the fewest in December/January and February. Significant effects on ‘sun-shade’ usages were also seen across the months (F_3, 878_ = 208.31, p < 0.0001; Fig 6). The fewest hens were found to use the ‘sun-shade’ in December/January which then linearly increased across the months. Hens’ distribution on the range in the ‘cloud’ varied across the months (F_3, 674_ = 7.13, p < 0.001; Fig 6). The most hens were observed in the ‘cloud’ during April and the fewest in February. Month also affected hens in the ‘cloud-shade’ (F_3, 539_ = 83.13, p < 0.0001; Fig 6) with the fewest hens observed to use the ‘cloud-shade’ in February and the most in April (Fig 6).

#### Relationship between sunlight and hens’ range use

**Fig 7:**
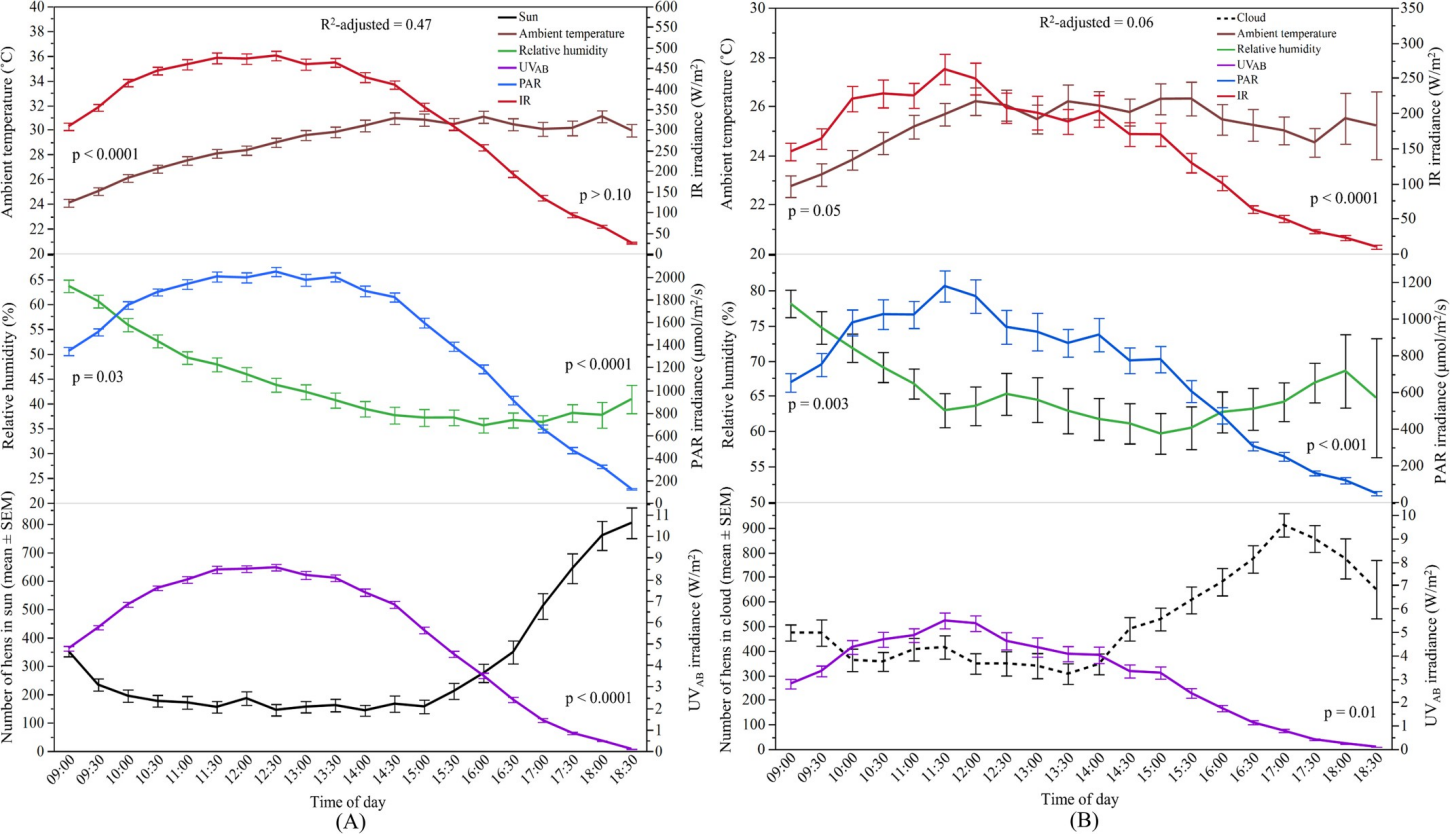
Relationship between sunlight spectrums and weather variables with the number of hens in the ‘sun’ and ‘cloud’ in QLD: (A) Y-axis (left): The mean (±SEM) number of hens in the ‘sun’, and the mean (±SEM) relative humidity and ambient temperature; Y-axis (right): the mean (±SEM) irradiance of UV_AB_ (ultraviolet radiation A and B wavelengths), PAR (photosynthetically active radiation), and IR (infrared radiation); (B) Y-axis (left): The mean (±SEM) number of hens in the ‘cloud’, and the mean (±SEM) relative humidity and ambient temperature; Y-axis (right): the mean (±SEM) irradiance of UV_AB_ (ultraviolet radiation A and B wavelengths), PAR (photosynthetically active radiation), and IR (infrared radiation). p > 0.10 indicates the variable had no significant effect and was removed from the final model.

The relationship of the predictors with ranging behaviour of hens in the ‘sun’ has been illustrated in Fig 7. The overall model was significant with ambient temperature, relative humidity, UV_AB_, and PAR as contributing factors (F_3.50, 1041.19_ = 244.93, p < 0.0001) explaining 47% of the variance. The model indicated that more hens were in the ‘sun’ when all the associated predictors decreased. PAR accounted for 34.4% of the variation in the model, followed by UV_AB_ (31.4%), ambient temperature (31%) and relative humidity (3.7%). For hens in the ‘cloud’, the best-fitted overall model explained 6% of the variance with all the predictors in the model (F_3.71, 692.81_ = 15.20, p < 0.0001). Most of the predictors had a negative relationship with the number of hens in the ‘cloud’ except IR, which was positively associated.

The effects of the predictors on hen ranging patterns in the ‘sun’ across the months are presented in Table 5. For the number of hens in the ‘sun’, the greatest variance (63%) explained by the sunlight and weather variables was in December/January where ambient temperature, UV_AB_ and PAR all showed a strong negative relationship. However, among the predictors, the two sunlight variables explained more than 90% of the variation with almost equal contribution to the model. The second largest explained variance (47%) was found in February with ambient temperature and UV_AB_ strongly negatively associated, and relative humidity showed a positive trend. The explained model variance reduced in March and April where predictors of ambient temperature, relative humidity and PAR all showed negative relationships.

**Table 5:** Multiple ridge regression analyses (ridge parameter, k = 0.02) on the number of hens in the ‘sun’ across the day in QLD for different observation months. Only variables that significantly contributed to the most parsimonious model are presented.

Month(s)	Predictor^1^	β-coefficient (Standardised)^ǂ^	t-value	P-value	Model’s F-statistics	Relative weight of the predictors in the model
December/January	Ambient temperature	-0.26	-8.28	< 0.0001	R^2^-adjusted = 0.63 F_2.48, 356.24_ = 217.73, p < 0.0001	9.1%
	UV_AB_	-0.46	-5.76	< 0.0001		45.1%
	PAR	-0.31	-3.91	< 0.0001		45.9%
February	Ambient temperature	-0.44	-6.50	< 0.0001	R^2^-adjusted = 0.47 F_2.89, 235.01_ = 76.82, p < 0.0001	36.8%
	Relative humidity	0.12	1.77	0.08		19.0%
	UV_AB_	-0.47	-10.34	< 0.0001		44.2%
March	Ambient temperature	-0.56	-10.87	< 0.0001	R^2^-adjusted = 0.38 F_2.92, 307_ = 68.66, p < 0.0001	60.3%
	Relative humidity	-0.11	-2.03	0.04		6.0%
	PAR	-0.36	-8.32	<0.0001		33.7%
April	Ambient temperature	-0.49	-5.95	<0.0001	R^2^-adjusted = 0.33 F_2.92, 135_ = 24.91, p < 0.0001	49.7%
	Relative humidity	-0.14	-1.71	0.09		5.1%
	PAR	-0.38	-5.63	< 0.0001		45.3%

^ǂ^β-coefficients (standardised) of the predictor variables were estimated separately using the ridge regression coefficient in ‘R’ as the original ridge package did not include the ‘β-coefficient’ value in the regression outputs.

^1^UV_AB_ (ultraviolet radiation A and B wavelengths), PAR (photosynthetically active radiation), IR (infrared radiation).

In the case of hens in the ‘sun-shade’, all the predictors influenced the hens’ distribution and described 29% of the variance (Table 6). Temperature was the most contributory predictor (76.3%) where temperature, relative humidity and PAR had a negative relationship, and both UV_AB_ and IR were positively associated with use of the ‘sun-shade’ areas (Table 6). For hens in the ‘cloud-shade’ the best-fitted overall model explained 21% of the variance with all the predictors included (Table 6). Similar to the ‘sun-shade’, there was a negative relationship with ambient temperature, relative humidity and PAR, and a positive relationship with UV_AB_ and IR indicating an increase in UV_AB_ and IR irradiance increased the number of hens in the ‘cloud-shade’ areas (Table 6).

**Table 6:** Multiple ridge regression analyses (ridge parameter, k = 0.02) on the number of hens in the ‘sun-shade’ and ‘cloud-shade’ across the day in QLD for all observation months. Only variables that significantly contributed to the most parsimonious model are presented.

Range distribution	Predictor^1^	β-coefficient (Standardised)^ǂ^	t-value	P-value	Model’s F-statistics	Relative weight of the predictors in the model
Sun-shade	Ambient temperature	-0.62	-18.09	< 0.0001	R^2^-adjusted = 0.29 F_4.35, 893.18_ = 85.02, p < 0.0001	76.3%
	Relative humidity	-0.24	-7.25	< 0.0001		8.7%
	UV_AB_	0.24	3.42	< 0.0001		3.1%
	PAR	-0.37	-4.82	< 0.0001		7.7%
	IR	0.24	3.56	< 0.0001		4.1%
Cloud-shade	Ambient temperature	-0.40	-6.95	< 0.0001	R^2^-adjusted = 0.21 F_3.77, 554.75_ = 41.07, p < 0.0001	11.1%
	Relative humidity	-0.57	-10.26	< 0.0001		39.9%
	UV_AB_	0.37	3.68	< 0.0001		13.4%
	PAR	-0.38	-4.27	< 0.0001		16.4%
	IR	0.32	3.45	< 0.0001		19.2%

^ǂ^β-coefficients (standardised) of the predictor variables were estimated separately using the ridge regression coefficient in ‘R’ as the original ridge package did not include the ‘β-coefficient’ value in the regression outputs.

^1^UV_AB_ (ultraviolet radiation A and B wavelengths), PAR (photosynthetically active radiation), IR (infrared radiation).

### Western Australia (WA)

The indoor shed on this farm was in a north-south direction where hens could range on both the east and west side. There were large trees located at the southeast corner with only one tree captured in the video recordings used for observations. The shade within the observation area was created by the shade of the indoor shed as well as the large tree and this varied across the day. The indoor shed’s shade increased in the range area over the day. This resulted in a reduction of the counting area for hens in the sun and a corresponding increase in the shaded areas until almost the whole counting area was shaded by the late afternoon.

#### Weather conditions and sunlight intensity

During the study period, the mean temperature and relative humidity was 24.3 ± 0.12˚C (ranged from 10.6 to 38.1˚C) and 48.8 ± 0.44% (ranged from 14.4 to 92.0%) respectively. The average sunlight irradiance was recorded as UV_AB_ irradiance 3.0 ± 0.06 W/m^2^ (ranged from 0.1 to 9.2 W/m^2^), PAR irradiance 1060.3 ± 16.9 μmol/m^2^/s (ranged from 10 to 2529 μmol/m^2^/s), and IR irradiance 256.0 ± 4.18 W/m^2^ (ranged from 0.30 to 593.2 W/m^2^). Both weather conditions and sunlight irradiance varied across the months as expected (Table 7).

**Table 7:** Mean daily (± SEM) outdoor conditions during ranging time across the months in WA (n = 67 days).

Variables^1^	January/February	March	April	May
Ambient temperature (˚C)	26.6 ± 0.30	25.5 ± 0.24	24.5 ± 0.18	21.9 ± 0.22
Relative humidity (%)	37.6 ± 1.14	52.3 ± 0.82	48.0 ± 0.67	53.1 ± 0.82
UV_AB_ (W/m^2^)	5.3 ± 0.14	3.7 ± 0.11	2.5 ± 0.08	2.0 ± 0.10
PAR (μmol/m^2^/s)	1665.0 ± 40.80	1253.8 ± 32.68	931.3 ± 24.04	780.9 ± 29.46
IR (W/m^2^)	379.4 ± 10.54	293.5 ± 8.45	229.9 ± 6.22	200.3 ± 7.62

^1^UV_AB_ (ultraviolet radiation A and B wavelengths), PAR (photosynthetically active radiation), IR (infrared radiation).

#### Effects of time of day on hens’ distribution outside

**Fig 8:**
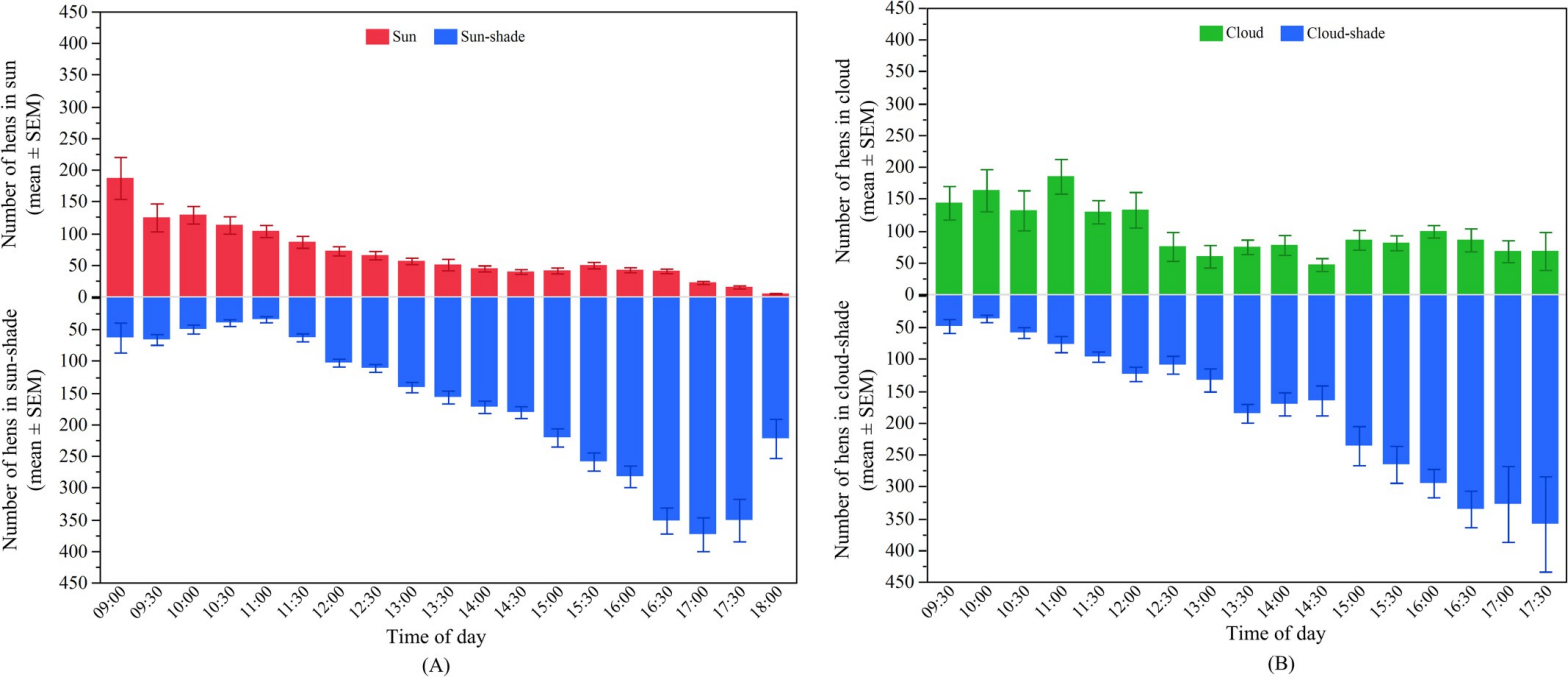
Distribution of hens on the range across the day in WA: (A) The mean (± SEM) number of hens in the ‘sun’ (top) and ‘sun-shade’ (bottom); (B) The mean (± SEM) number of hens in the ‘cloud’ (top) and ‘cloud-shade’ (bottom).

Time of day had effects on the hens’ distribution in both the ‘sun’ (F_18, 729_ = 14.00, p < 0.0001) and in the ‘sun-shade’ (F_18, 729_ = 158.70, p < 0.0001; Fig 8). Generally, a higher number of hens were observed in the ‘sun’ between 09:00 and 11:30 and then steadily decreased until the evening (p < 0.003; Fig 8). The opposite pattern was observed for hens ranging in the ‘sun-shade’ with fewer hens between 09:00 and 11:30 followed by a gradual increase up to 17:30 (p < 0.003; Fig 8).

The hens’ distribution in both ‘cloud’ and ‘cloud-shade’ also varied across the day (‘cloud’: F_16, 152_ = 3.74, p < 0.0001; ‘cloud-shade’: F_16, 152_ = 31.44, p < 0.0001, Fig 8). There were more hens in the ‘cloud’ across the morning (p < 0.003; Fig 8), whereas the hens ranging in the ‘cloud-shade’ showed a linear increase across the day (p < 0.003; Fig 8).

#### Differences in hens’ distribution across months

**Fig 9:**
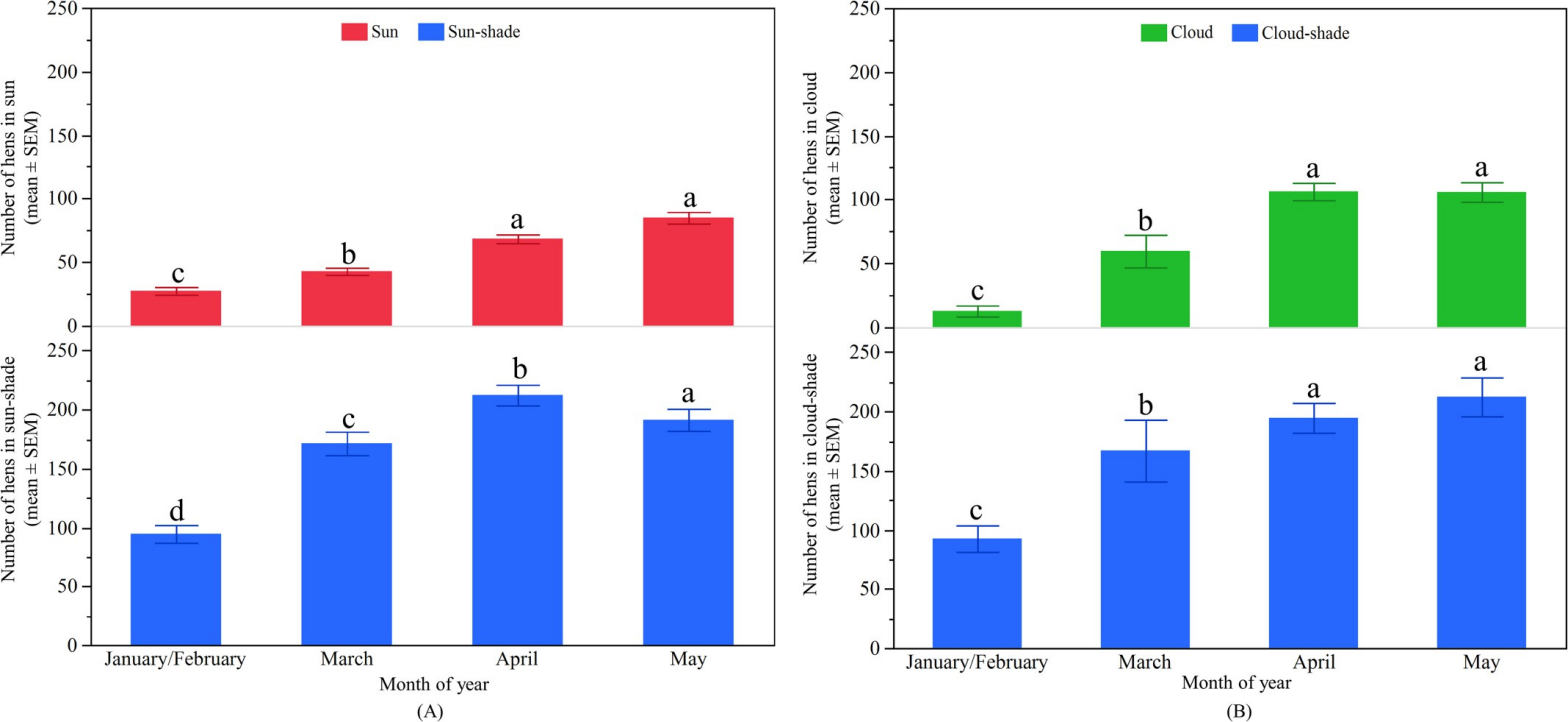
Distribution of hens on the range across months in WA: (A) The mean (± SEM) number of hens in the ‘sun’ (top) and ‘sun-shade’ (bottom); (B) The mean (± SEM) number of hens in the ‘cloud’ (top) and ‘cloud-shade’ (bottom). ^a-d^ Dissimilar superscript letters indicate significant differences among months. Raw values are presented with analyses conducted on transformed data.

There was significant variation between the months for hens ranging in the ‘sun’ (F_3, 729_ = 60.68, p < 0.0001) as well as in the ‘sun-shade’ (F_3, 729_ = 289.98, p < 0.0001; Fig 9). The number of hens venturing into the ‘sun’ and ‘sun-shade’ linearly increased across the months, but no difference was found in the ‘sun’ between April and May (Fig 9). Hens in the ‘cloud’ (F_3, 152_ = 30.45, p < 0.0001) and the ‘cloud-shade’ (F_3, 152_ = 34.32, p < 0.0001) also varied across the months with similar patterns of increases in hen numbers as the months progressed (Fig 9).

#### Relationship between sunlight and hens’ range use

**Fig 10:**
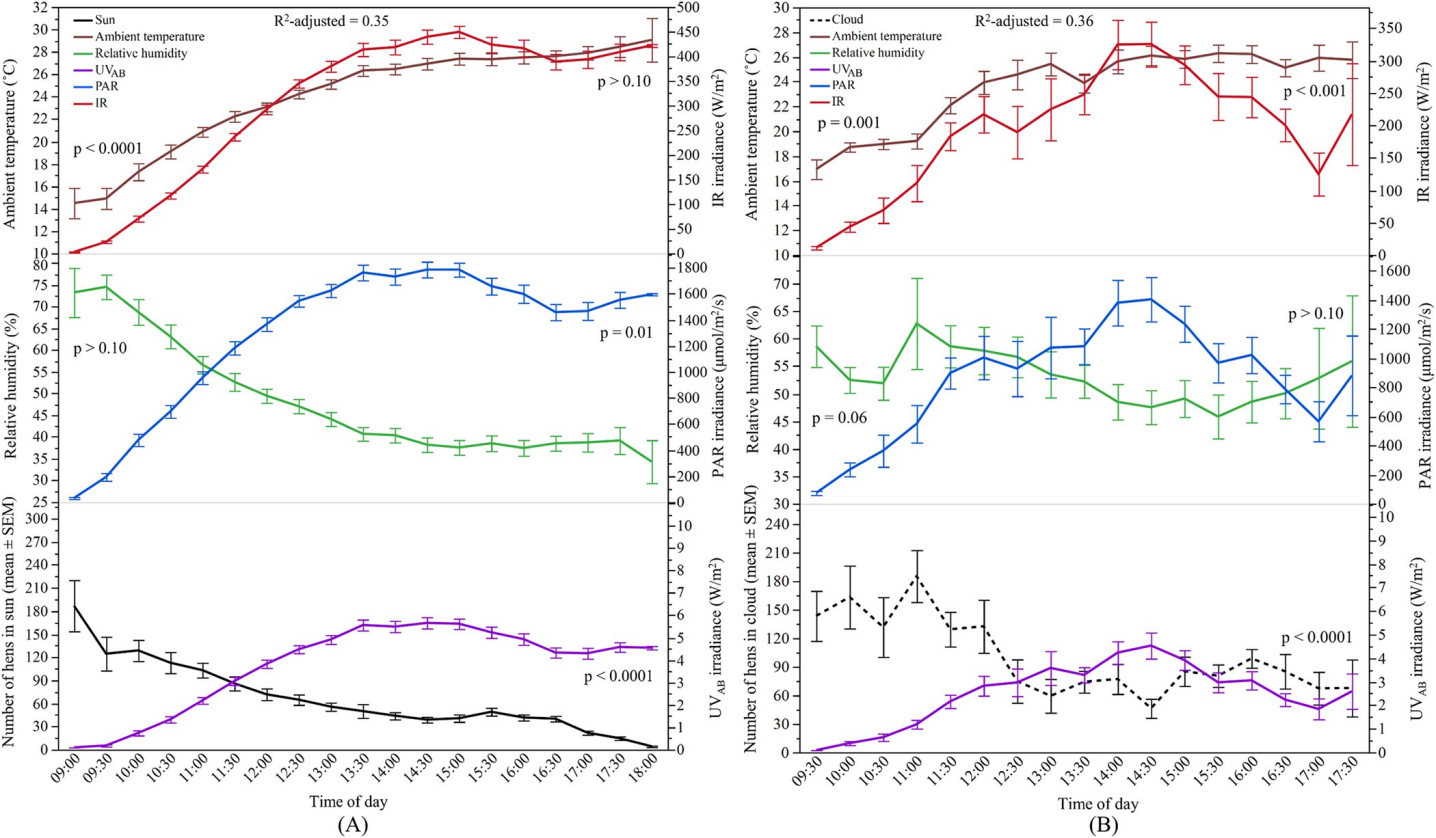
Relationship between sunlight spectrums and weather variables with the number of hens in the ‘sun’ and ‘cloud’ in WA: (A) Y-axis (left): The mean (±SEM) number of hens in the ‘sun’, and the mean (±SEM) relative humidity and ambient temperature; Y-axis (right): the mean (±SEM) irradiance of UV_AB_ (ultraviolet radiation A and B wavelengths), PAR (photosynthetically active radiation), and IR (infrared radiation); (B) Y-axis (left): The mean (±SEM) number of hens in the ‘cloud’, and the mean (±SEM) relative humidity and ambient temperature; Y-axis (right): the mean (±SEM) irradiance of UV_AB_ (ultraviolet radiation A and B wavelengths), PAR (photosynthetically active radiation), and IR (infrared radiation). p > 0.10 indicates the variable had no significant effect and was removed from the final model.

The relationship of the predictors with ranging behaviour of hens in the ‘sun’ is illustrated in Fig 10. The overall model was significant with ambient temperature, UV_AB_, and PAR as contributing factors (F_2.55, 748.16_ = 149.08, p < 0.0001) explaining 35% of the variance. The ambient temperature and UV_AB_ contributed 39.4% and 33.6% respectively to the model and had a negative relationship with hens in the ‘sun’, while IR had a positive relationship (27%). For hens in the ‘cloud’, ambient temperature and UV_AB_ showed a negative relationship while relative humidity and IR had a positive relationship. These contributory variables together explained 36% of the variance in the model (F_3.71, 170.05_ = 29.28, p < 0.0001; Fig 10).

The model effects across different months are presented in Table 8. However, there were only 9 days of available data together from January and February which may limit the interpretations of the model. Nevertheless, the model showed that ambient temperature and UV_AB_ were negatively associated with the number of hens in the ‘sun’. In March and April, the models showed ambient temperature, UV_AB_ and PAR accounted for 38% and 19% of the variation respectively, with ambient temperature and UV_AB_ showing a negative relationship, and PAR showing a positive relationship with hens in the ‘sun’. The model in May explained 16% of the variation where only the weather variables rather than the sunlight variables significantly contributed to the model.

**Table 8:** Multiple ridge regression analyses (ridge parameter, k = 0.02) on the number of hens in the ‘sun’ across the day in WA for different observation months. Only variables that significantly contributed to the most parsimonious model are presented.

Month(s)	Predictor^1^	β-coefficient (Standardised)^ǂ^	t-value	P-value	Model’s F-statistics	Relative weight of the predictors in the model
January/February	Ambient temperature	-3.32	-4.16	< 0.0001	R^2^-adjusted = 0.39 F_2.75, 126.05_ = 34.62, p < 0.0001	43.7%
	UV_AB_	-0.61	-4.60	< 0.0001		17.6%
	IR	-0.82	-5.63	< 0.0001		38.7%
March	Ambient temperature	-0.48	-7.22	< 0.0001	R^2^-adjusted = 0.38 F_2.65, 165.10_ = 40.91, p < 0.0001	57.0%
	UV_AB_	-0.64	-4.46	< 0.001		27.1%
	PAR	0.46	3.33	0.001		15.9%
April	Ambient temperature	-0.24	-3.69	< 0.001	R^2^-adjusted = 0.19 F_2.58, 274.14_ = 24.26, p < 0.0001	36.5%
	UV_AB_	-0.48	-3.57	< 0.001		37.4%
	PAR	0.24	1.78	0.08		26.1%
May	Ambient temperature	-0.21	-2.35	0.02	R^2^-adjusted = 0.16 F_1.93, 175_ = 17.58, p < 0.0001	48.3%
	Relative humidity	0.23	2.60	0.01		51.7%

^ǂ^β-coefficients (standardised) of the predictor variables were estimated separately using the ridge regression coefficient in ‘R’ as the original ridge package did not include the ‘β-coefficient’ value in the regression outputs.

^1^UV_AB_ (ultraviolet radiation A and B wavelengths), PAR (photosynthetically active radiation), IR (infrared radiation).

For the number of hens in the ‘sun-shade’, all predictor variables except relative humidity were significant (Table 9), accounting for 36% of the variance. UV_AB_ and PAR were negatively associated with hens in the ‘sun-shade’, and ambient temperature and IR were positively associated. However, IR (61.2%) and PAR (22.6%) showed the greatest contributions in the model. All the predictors in the ‘cloud-shade’ model were significant where UV_AB_ and PAR had a negative relationship and rest of the variables showed a positive relationship with the number of hens in the ‘cloud-shade’ (Table 9).

**Table 9:** Multiple ridge regression analyses (ridge parameter, k = 0.02) on the number of hens in the ‘sun-shade’ and ‘cloud-shade’ across the day in WA for all observation months. Only variables that significantly contributed to the most parsimonious model are presented.

Range distribution	Predictor^1^	β-coefficient (Standardised)^ǂ^	t-value	P-value	Model’s F-statistics	Relative weight of the predictors in the model
Sun-shade	Ambient temperature	0.16	5.13	< 0.0001	R^2^-adjusted = 0.36 F_3.31, 747.24_ = 235.05, p < 0.0001	6.5%
	UV_AB_	-0.44	-6.78	< 0.0001		9.7%
	PAR	-1.19	-16.70	< 0.0001		22.6%
	IR	1.72	28.42	< 0.0001		61.2%
Cloud-shade	Ambient temperature	0.55	7.0	< 0.0001	R^2^-adjusted = 0.24 F_4.19, 169.33_ = 20.67, p < 0.0001	32.8%
	Relative humidity	0.27	3.96	< 0.0001		8.2%
	UV_AB_	-0.66	-4.19	< 0.0001		9.1%
	PAR	-0.63	-3.59	< 0.0001		14.9%
	IR	1.22	7.89	< 0.0001		35.0%

^ǂ^β-coefficients (standardised) of the predictor variables were estimated separately using the ridge regression coefficient in ‘R’ as the original ridge package did not include the ‘β-coefficient’ value in the regression outputs.

^1^UV_AB_ (ultraviolet radiation A and B wavelengths), PAR (photosynthetically active radiation), IR (infrared radiation).

## Discussion

The aim of this study was to understand the relationship between hen range use on commercial farms within different regions of Australia and different wavelengths of sunlight across the summer/autumn months. This study confirmed previously observed variation in ranging behaviour across the day with fewer hens outside in direct sunlight during the midday period. The variables of ambient temperature, relative humidity, UV_AB_ (ultraviolet A and B wavelengths), PAR (photosynthetically active radiation) and IR (infrared radiation) all impacted hen ranging to varying degrees across both study months and farm sites. Both the climatic variation and structural layout between farms may have contributed to the differences between the three study flocks. These results support the necessity for adequate shelters in the range area to facilitate use of the outdoor areas in countries that experience intense solar radiation as well the actions to be taken in response to increasing temperatures due to climate change globally.

### Time of day and month effects

The pattern of hen ranging behaviour was distinct between sunny and cloudy conditions and supported previous studies showing more hens ranging outside during cloudy and dull weather compared to intense sunny days [43, 44]. Moreover, there were clear time of day effects on hens’ range occupancy under both direct sunlight and sun-shaded areas. Similar to previous research [15, 20, 21], more hens were seen in the sun in the morning which then gradually decreased as the day progressed until the maximum number of hens were found in the sunshine part of the range in the late afternoon/evening. A corresponding opposite pattern was seen for the hens in sun-shaded portions of the range. These patterns were particularly clear in TAS and QLD where hens had ample range area under direct sunlight throughout the day. In contrast, the sun part of the range decreased over the day in WA highlighting the effect that shed orientation can have on the ranging environment, and thus, patterns of range use across the day. While previous studies have assessed the total percentage of hens ranging outside rather than distinguishing between hens in direct sunlight or shade within the range [9, 15, 20, 21], other supporting research has shown greater use of shaded/covered areas during the midday period relative to the morning and late afternoon [17, 45].

Similar to the time-of-day effects, this study showed increasing use of sun areas of the range as the months progressed from summer (commences December 1) through to autumn (commences March 1). This result corresponds with that of Hegelund et al. [20] who observed a strong seasonal effect on hens ranging outside with more hens using the outdoor range in autumn and fewer in the spring and summer seasons in Denmark. Correspondingly, Nagle and Glatz [17] observed a six-fold increase in use of the overhead shaded areas versus non-shaded areas in the summer by hens in Australia.

### Temperature and relative humidity

Both temperature and relative humidity significantly affected hens’ distribution on the range but with temperature having a much greater impact across all farms and all months of observation. Physiologically, hens’ thermoneutral zone within an optimal surrounding temperature is typically between 20˚C and 25˚C [46]. However, the response to environmental temperature could be varied with genetic strains and adaptation processes [47]. Temperatures above the optimal range activates birds’ thermoregulatory mechanisms by increasing the respiratory rate and loss of heat through evaporation [48]. In commercial free-range farm settings in Denmark, the effects of temperature on hens’ range use was demonstrated by Hegelund et al. [20] who found that the maximum number of hens used the range with ambient temperatures around 17°C, after which range use gradually decreased. Richards et al. [21] observed a clear linear relationship indicating the use of the range on a UK commercial farm increased with rises in temperature up to around 20°C. These results support the negative relationships found in the current study where the mean recorded temperatures were 21.2°C, 27.2°C, and 24.3°C in TAS, QLD and WA, respectively and the maximum temperature was much higher than those expected in European countries. The range areas under direct sunlight likely exceeded the heat tolerance of hens resulting in preferences for shaded and likely cooler areas. This was evident in the relatively higher occupancy of the ‘sun-shaded’ and ‘cloud-shaded’ areas for TAS and WA in the present study, indicating hens will seek out shade as the temperature under direct sunlight increases. Future research measuring the temperature in the shaded areas may clarify why there was a negative association between ambient temperature and hens in both the ‘sun-shade’ and ‘cloud-shade’ areas on the QLD farm.

The effect of relative humidity on hens’ range distribution was more varied showing both positive and negative associations with range use depending on the farm and month and was typically not a large contributing factor in the models. Results across other studies have also varied. No effect of relative humidity was found on range use in one study in the UK [23], or more hens ranged away from the indoor shed when the humidity was low across another UK study [15]. Across farms in Denmark, fewer hens were outside when it was raining, but high atmospheric humidity saw similar numbers of hens outside to that observed on dry days [20]. In addition to relative humidity and temperature, other weather variables have been shown to have significant effects on range use such as wind speed, rainfall, and atmospheric pressure [15, 20, 21]. Measurement of all these additional variables may have further contributed to the explanatory power of the models. Similarly, environmental conditions within the indoor shed may have also affected whether hens go outside [8] which could be included in future assessments.

### Sunlight spectrums and intensity

Sunlight contains a range of radiations of which only three types reach the earth’s surface with the majority being IR (56%) and PAR (39%), and only 5% are UV_AB_ [49]. These different spectrums were all predicted to influence hens’ range use to varying degrees. Overall, each spectrum did have a significant impact on range distribution of hens in the direct sunlight or shaded areas, but the strength and direction of the relationships varied across farms and across months. The effects of both UV_AB_ and PAR were more evident in QLD (contribution in the model 31% and 34% respectively, total 65% for hens in the ‘sun’) which might be due to higher mean irradiances of both spectrums on this farm relative to the other two study farms. Additionally, in the overall model for TAS, UV_AB_ did not have a significant impact on hens’ range use in the ‘sun’ highlighting how region may affect typical ranging patterns.

Typically, PAR and UV_AB_ spectrums were strongly negatively correlated with the hens in the direct sunlight, indicating that as these irradiances increased, hens either moved into the shade or remained inside the shed. This was expected based on the predicted aversiveness of visibly bright sunlight and the damaging effects of high ultraviolet radiation. These results are similar to previous studies that have shown slow-growing broilers will reduce ranging and increase their use of shade when total solar irradiance increases [26, 50], although the irradiance in these studies was not categorised into different wavelengths. The results also align with previous observations of free-range hens and broilers where fewer birds were outside on days visually categorised by researchers as ‘bright’ [24, 51]. Correspondingly, during the hen counts in this study, more birds were observed outside on cloudy days, similar to an observation reported by Whay et al. [44] during visits to 25 commercial free-range farms in the UK where they noticed that maximum use of the range happened on calm, cloudy days. UV supplementation in indoor settings can improve hens’ behavioural repertoire and skeletal heath [7] and hens will show preferences for low levels of UV_AB_ light compared to UV deficient environments [33, 52]. This may support the birds’ greater use of the ‘sun’ range area in the morning and late afternoon, but when the radiation is at its peak irradiance, hens avoid this, similar to hens exhibiting preferences for shelters that block more UV radiation [34–36]. As chickens can see in the UV spectrum [53, 54], they may have also been avoiding the peak UV_AB_ radiation because it was visually aversive. Further on-farm observations paired with solar radiation sensors that detect A and B wavelengths separately would confirm this.

In contrast to PAR and UV_AB_, overall, IR had a positive relationship with the number of hens in the ‘sun’ in TAS as well as hens in the ‘cloud’ and ‘cloud-shade’ for both QLD and WA; however, similar to other wavelengths, the presence and strength of the relationship varied across the months. IR is the light spectrum that has the most influence on environmental temperature. The earth’s surface absorbs heat from IR, which is then converted into thermal energy raising surrounding temperatures [37]. Ambient temperature had stronger effects on hens’ range use than specifically IR wavelengths, and sometimes the direction of impact was inverse. It is possible that hens may use IR under direct sunlight to warm up, but further work is needed to better understand the relative effects of IR and temperature on hens’ thermal comfort. While sunlight wavelengths, temperature and humidity showed clear relationships with hens’ range use on the commercial farms, these effects were strongest under direct sunlight. There were significant relationships between the predictors and hens’ occupancy of the shaded range areas but the R^2^ values were very low indicating they did not explain much of the observed variation. Range enrichment of adequate shelters with large canopy covers will likely have benefits in protecting hens from the adverse effects of high temperatures and intense sunlight.

## Conclusion

To the best of the authors’ knowledge, this is the first study to investigate the impacts of different wavelength ranges of sunlight on hen ranging patterns across different commercial farms without altering any resources in the indoor shed or on the outdoor range. This study shows that hens are sensitive to specific wavelengths of sunlight, and they avoid intense radiation that may be visually aversive or damaging. Hens will also avoid direct sunlight if the temperatures are high and may cause heat stress. There will be variation across seasons as to the degree of sunlight influence, as well as between regions. However, only a single shed on each farm was observed per region so some of the variation in results may be attributable to the sample selection. Overall, the sunlight and selected weather parameters only accounted for part of the variation in range usage indicating that there are other factors that will affect hens’ decision to range. The hens’ aversion to strong sunlight during the summer months highlights the need for adequate shade on the range that may increase hens’ ranging time outside.
